# Cincinnati Academy of Medicine

**Published:** 1881-08

**Authors:** 


### CINCINNATI ACADEMY OF MEDICINE.

### CANCRUM ORIS.

Dr. Cleveland reported a case of gangrene of the mouth (cancrum oris) that had occurred in his practice. It occurred in a little child, æt. two years. The child had been sick for some three months with chronic pneumonia and was apparently improving, the fever had subsided and the child seemed to be gaining strength, when a high fever set in with great prostration. The little patient emaciated rapidly and became very feeble. Her bowels were very costive and stomach irritable; for this condition a purgative dose of calomel was given. In two days afterwards salivation was noticed, with puffing of the gums and lips and loosening of the incisors above and below. Commencing with the gums of the incisor teeth the gangrene (for such it proved to be) rapidly extended. It attacked the end of the tongue, but showed no disposition to extend further, nor did it extend in the upper maxilla beyond the first region of attack (around the superior incisors). The superior incisor teeth fell out and the alveola was exposed and black, but below the ravages were extensive, the incisor gums were destroyed and it extended over the lower lip by way of the mucous membrane, destroyed the lower lip in its entirety from the corners of the mouth, making a crescent sweep two-thirds of the way to the edge of the chin. Death ended the scene on the ninth day of the attack. The child was so debilitated and it was so evident that it could not live that heroic measures, like the actual cautery and nitric acid, were not considered, palliative measures alone were resorted to. As is usual in these cases, the disease appeared to progress without pain. Also, as is always the case, the disease was secondary, the patient being broken down by a previous disease. The hygienic surroundings in this case were in every respect favorable. This is contrary to the rule, as it is a disease usually occurring in foundling hospitals and among the feeble and poor. Mercury was regarded as a possible exciting cause of the disease, but that this was what developed it was not looked upon as at all probable. Measles were present in the house at the time and the possibility of this being the exciting cause was suggested. Some English statistics go to show that measles precede quite a noticeable number of cases of gangrene.

DISCUSSION.

Dr. Whittaker, in opening the discussion, remarked that the disease is of comparatively rare occurrence and even then is not infrequently overlooked. He had seen but one case and that was in the Cincinnati Hospital. The name covers many diseases, but as here understood means noma or gangrene of the mouth and is distinguished from apthous, catarrhal or ulcerative stomatitis, by being attended with necrosis of tissue. What is the cause of it and why the face should be specially subject to this disease in young children is not known. It is never primary, but secondary to some infectious malady that is attended with coagulation of the blood. All authorities agree that measles is the primary malady in nine tenths of all the cases. The black point speaks for loss of nutrition. The speaker did not believe that mercury, as frequently supposed, was a cause of cancrum oris. It is given in all sorts of affections as a laxative, and if it were the offending agent the disease in question ought to be of much more frequent occurrence. Ptyalism may be produced, but when the drug is checked at this time, there is no fear of noma. It is only when given for a long time in small doses frequently repeated that we might find a plausible explanation in attributing the disease to the use of mercury, but not when given for its laxative effect only. The child in the instance reported certainly had some infectious malady, probably a masked measles, but possibly of a pulmonary character, though the essayist did not state what it was. The disease did not depend on any external cause in the majority of cases, but came from within and depended upon an occlusion of a nutrient vessel causing gangrene. As regards treatment not much can be said; cauterization with nitric acid limits the spread of it and consequently ought to be practiced when the disease is seen early enough. During the war great praise was sung of bromine applied pure as the best caustic and antiseptic.

Dr. Tate, in answer to a question, replied that he never saw this disease as a result from the use of mercury. He saw two cases of noma in which the trouble commenced in the mucous membrane of the cheek and then followed the usual course. The case reported differed from the usual form inasmuch as the mucous membrane adjoining the gums was the origin of the disease, an inflammatory affection characterized by profuse secretion seizing the cheek and then destroying not only the gums but proceeding inwards and causing necrosis of bone. This history with the great prostration of the general system as in measles in the great majority of instances result from a depravity of the blood. But the question may be asked if the patient in the case reported had had no mercury would he have had noma? Is it not possible that mercury was the exciting cause inducing this unfortunate result? The speaker could not prove from personal observation, but it seemed to him as if noma was not now so frequent as when calomel was more freely used and salivation was more common. It was remarkable that in the case cited the hygienic surroundings were good, generally they are unfavorable; both of the speaker's cases were of a depraved constitution, one breathing in the foul air of a damp cellar, the other following after a long course of typhoid fever.

The treatment consists in upholding the system and cauterizing the ulceration, preferably, in Dr. Tate's opinion, with strong nitric acid. There is no question, that although the disease is very fatal, a number of cases get well.

Dr. Nickles never had a case of gangrene of the mouth, but some years ago was called to a coroner's inquest on a child, five years of age, to whom a quack had given calomel in excessive doses. The speaker, however, as well as another physician, concluded that it was not the calomel that caused the disease.

Dr. Drury said that he saw Dr. Cleveland's case in the last days. The Doctor told him he had given calomel and thought that salivation might have caused the trouble. The mother or nurse first observed the condition of the mouth, beginning with a swelling, then the spot, etc. This process probably began in the mucous membrane of the mouth or the muscular tissue beneath. Measles is said to be a frequent cause of the disease, one author reporting thirty-six out of forty-eight, another forty-one of sixty-eight cases of gangrene of the mouth and measles respectively. As regards treatment English authorities recommend the use of strong nitric acid, applied with a pledget of lint and thoroughly rubbed in the gangrenous part.

Dr. Cleveland, in conclusion, stated that measles existed in the house in which he had his case, though the child had no eruption, if it had the disease. He was at a loss to account what the pulmonary disease was; there was consolidation of lower lung, tubular respiration, later emaciation and death. It may have been tuberculosis or chronic pneumonia. Convalescence had taken place before the relapse, which fired up the pulmonary symptoms. Treatment was out of the question because death was inevitable. He would have cauterized with the hot iron or strong nitric acid if there had been any hope, but in this instance it would have been futile.

*Lancet and Clinic.*

---

To destroy the odor of foul breath—The smell of the axilla and the fetor of the sweat of the feet:

℞ Potass. permanganat.........................gr. vj.
Aquæ.............................................oz. vj.

Sig.—Apply frequently.

It is a fact too little appreciated by physicians and dentists that success in practice often depends more on attending to some such trivial affection as the above, than on the successful management of a complicated case.

The Mortality from Anæsthetics in Great Britain, for the decade ending 1880, was as follows; Chloroform 101; ether 11; Chloroform and ether, 7; methyleno 10.—*British Med. Journal.*

